# Chronic bronchitis in West Sweden – a matter of smoking and social class

**DOI:** 10.3402/ecrj.v3.30319

**Published:** 2016-07-13

**Authors:** Malin Axelsson, Linda Ekerljung, Jonas Eriksson, Stig Hagstad, Eva Rönmark, Jan Lötvall, Bo Lundbäck

**Affiliations:** 1 Department of Care Science, Faculty of Health and Society, Malmö University, Malmö, Sweden; 2 Department of Internal Medicine and Clinical Nutrition, Krefting Research Centre and Environmental and Occupational Medicine, University of Gothenburg, Gothenburg, Sweden; 3 Halland Region, Kungsbacka Health Centre, Kungsbacka, Sweden; 4 Research and Development, Obstructive Lung Disease In Northern Sweden (OLIN) Studies, Norrbotten County Council, Luleå, Sweden; 5 Department of Public Health and Clinical Medicine, Division of Occupation and Environment/The OLIN Unit, University of Umeå, Umeå, Sweden

**Keywords:** chronic bronchitis, epidemiology, population study, respiratory tract disease, risk factors

## Abstract

**Background:**

Although chronic bronchitis is associated with impaired quality of life, hospitalisations and increased mortality, it has been less in focus after the introduction of the term chronic obstructive pulmonary disease (COPD). There are no recent published data on the prevalence of chronic bronchitis from the Scandinavian countries.

**Aim:**

The main aim of the present study was to estimate the prevalence of chronic bronchitis in West Sweden by using data from a large-scale epidemiological study of the general population. A further aim was to identify current risk factors for chronic bronchitis in a population with a major decrease in the proportion of smokers.

**Methods:**

From the 18,087 questionnaire responders out of 30,000 invited to participate at the West Sweden Asthma Study, 2,000 subjects were randomly selected and invited to detailed clinical examinations performed during 2009–2013. A total of 1,172 subjects aged 17–79 participated in the examinations which included, among others, spirometry and structured interviews. Chronic bronchitis was defined according to reported symptoms.

**Results:**

The overall prevalence of chronic bronchitis was 7.2% (men 7.6%; women 6.8% ns), and it was 8.7% in subjects older than age 60. Chronic bronchitis was strongly associated with smoking, defined both as current smoking status and pack-years. Other risk factors were increasing age, low socio-economic class and urban living. Of those with chronic bronchitis, 22% fulfilled the GOLD criteria of COPD.

**Conclusion:**

The prevalence of chronic bronchitis was somewhat lower than found by studies in Sweden in the 1980s and the prevalence was now similar in men and women. Although smoking was still the dominating risk factor for chronic bronchitis, the relative importance of smoking had decreased parallel with a decreasing smoking prevalence, while the relative importance of other factors than smoking had increased compared to previous studies.

Chronic bronchitis is a common disease with no major change of its definition after the CIBA Guest Symposium in 1959 (1). However, it has been less in focus after the introduction of the term chronic obstructive pulmonary disease (COPD), which from the 1960s has been increasingly used. Major guidelines for the management of COPD were developed during the 1990s (2–4). Although chronic bronchitis and COPD often coexist (5–7), it is important to remember that they are two different diagnoses (7). The major symptoms of chronic bronchitis are chronic mucus hyper-secretion often accompanied by cough (1). Chronic bronchitis often precedes COPD (8, 9) and also occurs in individuals with normal lung function (7). Chronic bronchitis is associated with anxiety and depression (10), poor quality of life (11), accelerated decline in lung function (12–14) and increased mortality (12, 14–16). Large-scale epidemiological studies have shown chronic bronchitis without COPD to be even more associated with premature death than mild COPD (16). The major risk factor for COPD in the Western world is smoking (17–20), and other important risk factors are air pollution (21, 22) and occupational airborne exposures (15, 19, 23).

The global prevalence of chronic bronchitis has been estimated at 6.4% (24), and the prevalence of chronic bronchitis varies considerably by age, sex and geographical area from 2.3 to 28.2% (17–19, 25–28). According to the Obstructive Lung Disease in Northern Sweden (OLIN) studies, in Sweden, the prevalence during the 1980s was 10.4% in men and 7.4% in women (17). Recently, in the Respiratory Health in Northern Europe (RHINE) studies, which involves the North European part of the European Community Respiratory Health Survey II (ECRHS II), the estimated prevalence of chronic bronchitis was 5.4% in young to middle-aged adults (19).

Considering that tobacco smoking is a significant risk factor for the development of chronic bronchitis and that tobacco smoking has decreased in recent years in several parts of, for instance, Europe (27, 28) and Sweden (29, 30), and that there are, in contrast to COPD, few recently published data on the prevalence of chronic bronchitis, an update on the prevalence is warranted. The main aim of the present study was to estimate the prevalence of chronic bronchitis in West Sweden by using data from a large-scale epidemiological study of the general population. A further aim was to identify risk factors for chronic bronchitis in a population with a major decrease in the proportion of smokers.

## Material and methods

### Study sample

The study sample was derived from the population-based West Sweden Asthma Study (WSAS), which has been thoroughly described elsewhere (30). A good representativeness of the WSAS was concluded by a non-response analysis (31). Briefly, in 2008, 30,000 postal questionnaires were mailed to subjects aged 16–75 living in the region of West Gothia, south-west of Sweden. They were randomly selected from the Swedish population register. The response rate was 62%, and of the responders, 2,000 subjects were randomly selected to participate in clinical examinations from 2009 to 2013 including spirometry and structured interviews, and 1,172 attended (32). The current study is based on these 1,172 subjects (542 men, 46.2%) with a mean age of 49.9 years (SD 15.4). Additional background characteristics are presented in Table 1. The regional research ethics board at the University of Gothenburg approved the study.

**Table 1:** Background characteristics of the study sample by sex

	Men (*n*=542)	Women (*n*=630)		All
Variables	*N* (%)	*N* (%)	Difference (*p*)	*N* (%)
Age groups			**0.001**	
≤ 40 years	150 (27.7)	197 (31.3)		347 (29.6)
> 40 ≤ 60	194 (35.8)	276 (43.8)		470 (40.1)
> 60 years	198 (36.5)	157 (24.9)		355 (30.3)
Area of domicile			0.21	
Gothenburg	297 (54.8)	369 (58.6)		666 (56.8)
Västra Götaland	245 (45.2)	261 (41.4)		506 (43.2)
Level of education			**0.001**	
University	238 (43.9)	350 (55.6)		588 (50.2)
Lower than university	304 (56.1)	280 (44.4)		584 (49.8)
Current smoking status			0.25	
Non-smokers	264 (48.7)	297 (47.1)		561 (47.9)
Ex-smokers	199 (36.7)	220 (34.9)		419 (35.8)
Smokers	79 (14.6)	113 (17.9)		192 (16.4)
Pack-years among current smokers			0.84	
≤ 10 years	22 (27.8)	29 (25.7)		51 (26.6)
> 10 <20 years	5 (6.3)	25 (22.1)		30 (15.6)
≥ 20 years	28 (35.4)	36 (31.9)		64 (33.3)
Missing	24 (30.4)	23 (20.4)		47 (24.5)
Pack-years among ever smokers			**0.05**	
≤ 10 years	113 (40.6)	146 (43.8)		259 (42.4)
> 10 < 20 years	38 (13.7)	77 (23.1)		115 (18.8)
≥ 20 years	82 (29.5)	65 (19.5)		147 (24.1)
Missing	45 (16.2)	45 (13.5)		90 (14.7)
Exposure to occupational dust, gas or fumes			**0.001**	
No	344 (63.5)	532 (84.4)		876 (74.7)
Yes	195 (36.0)	93 (14.8)		288 (24.6)
Unknown	3 (0.6)	5 (0.8)		8 (0.7)

Difference (*p*-value) between men and women.

Bold values indicate significant differences.

### Data collection

Data were collected through a structured interview based on the Swedish OLIN questionnaire (33), which has been used in a large number of studies in Northern Europe and elsewhere (34–37) and has recently been validated (38). It includes questions about respiratory symptoms, diseases and medication for obstructive airway diseases, and further about background demographic characteristics including detailed questions about smoking habits and occupation. Lung function measurements were performed using a Jaeger spirometer and the Global Lungs Initiative (GLI) reference equation for spirometry was used (39).

### Definitions

Chronic bronchitis was defined as having sputum production when coughing on most days for at least 3 months during two subsequent years (1). Participants in the current study were classified as having chronic bronchitis, if they answered yes to the following question: ‘Do you bring up phlegm when coughing on most days during periods of at least 3 months per year?’ and answered more than 2 years on the following question: ‘If yes, for how many years?’

Participants were classified as having the respiratory symptom or lung disease, if they answered yes to the following questions:

Longstanding cough: ‘Have you had a longstanding cough during the last year?’

Sputum production: ‘Do you usually have phlegm when coughing, or do you have phlegm which is difficult to bring up?’

**A**ny wheeze: ‘Have you had whistling or wheezing in the chest at any occasion during the last 12 months?’

Recurrent wheeze: ‘Do you usually have whistling or wheezing when breathing?’

Wheeze most days periodically: ‘Do you have this whistling in your chest or wheezing most days a week, periodically?’

Persistent wheeze: ‘Do you have this whistling in your chest or wheezing most days a week?’

Wheeze apart from cold: ‘Have you had this wheezing or whistling in your chest when you have not had a cold?’

Wheeze with breathlessness: ‘Have you been at all breathless when you had wheezing or whistling in the chest?’

Asthmatic wheeze: a combination of ‘any wheeze’, ‘wheeze with breathlessness’ and ‘wheeze apart from cold’

Physician diagnosed chronic bronchitis: ‘Have you been diagnosed as having chronic bronchitis by a physician?’

Physician diagnosed COPD: ‘Have you been diagnosed as having chronic obstructive pulmonary disease by a physician?’

Physician diagnosed emphysema: ‘Have you been diagnosed as having emphysema by a physician?’

Currents smokers consisted of participants answering yes to the following question: ‘Do you smoke?’

Ever smokers consisted of subjects who were either current smokers or ex-smokers.

High level of education was defined as university education and low level of education was defined as high school, secondary school or less.

Exposure to occupational dust, gas or fumes was defined as answering yes to the following question: ‘Have you been heavily exposed to dust, gases or fumes at your work?’.

Dyspnoea grade was assessed using the Medical Research Council breathlessness scale (MRC scale) (40).

COPD was defined according to the criteria set by the Global Initiative for Chronic Obstructive Pulmonary Disease (GOLD) based on post-bronchodilator FEV_1_/FVC <0.70, and the disease severity was based on the GOLD spirometry criteria of FEV_1_, in percent of predicted (7).

### Analyses

All statistical analyses were performed using SPSS version 21. Fisher's exact test was used for comparisons of proportions, Mantel–Haenszel test was used to test trends and independent sample *t*-test was used for comparisons of mean values. A *p*-value of <0.05 was regarded as statistically significant. Bivariate and multivariate logistic regression models were used to identify factors associated with chronic bronchitis. Risks were expressed as odds ratios (OR) with 95% confidence interval (CI). A combined variable method was used to explore interactions of risk factors for chronic bronchitis.

## Results

### Prevalence of chronic bronchitis, respiratory symptoms and physician-diagnosed lung disease

The overall prevalence of chronic bronchitis was estimated at 7.2%, being somewhat higher in men (7.6%) than in women (6.8%), but the difference was not statistically significant. The prevalence increased significantly by age, being most common among subjects older than 60 years. No differences between men and women were found with regard to reported respiratory symptoms (Table 2). The prevalence of self-reported, physician-diagnosed chronic bronchitis was 3.5%, of physician-diagnosed COPD 1.6% and of physician-diagnosed emphysema 0.3% with no significant difference between men and women.

**Table 2:** Prevalence (%) of chronic bronchitis and respiratory symptoms by age groups and sex

	Age groups (years)				Sex			
			
Variables	≤40	>40 ≤60	>60	Difference (*p*)	Men	Women	Difference (*p*)	All
Chronic bronchitis	4.3	8.1	8.7	**0.028**	7.6	6.8	0.65	7.2
Long-standing cough	15.3	17.9	18.9	0.21	15.7	18.9	0.16	17.4
Sputum production	8.5	16.0	22.9	**0.001**	17.4	14.5	0.20	15.9
Any wheeze	22.5	24.3	18.9	0.26	21.2	22.9	0.52	22.1
Recurrent wheeze	5.8	10.4	10.4	**0.035**	9.2	8.9	0.84	9.0
Wheeze most days periodically	4.9	5.5	4.7	0.93	4.9	5.2	0.89	4.9
Persistent wheeze	0.3	4.3	3.2	**0.025**	2.9	2.7	0.86	2.6
Wheeze apart from cold	16.7	18.1	14.1	0.36	16.8	16.2	0.81	16.5
Wheeze with breathlessness	14.7	12.8	7.6	**0.004**	10.0	13.3	0.08	11.8
Asthmatic wheeze	11.0	9.8	5.9	**0.021**	8.3	9.5	0.47	9.0
Dyspnoea grade ≥ 2^a^	1.7	3.2	6.7	**0.001**	3.2	4.3	0.36	3.8
Dyspnoea grade ≥ 3^a^	–	0.2	2.6	**0.001**	0.7	1.0	0.76	0.9

Difference (*p*-value) between age-groups tested with Mantel–Haenszel test for trend; difference (*p*-value) between men and women tested with Fisher's exact test.

a MRC scale=Medical Research Council breathlessness scale: Grade 2=‘walk slower than most people my age on the level’ and/or ‘have to stop for breath when walking at my own pace on the level’. Grade 3=‘stop for breath after walking 100 yards on level ground’. Grade 4=‘get out of breath when I wash myself or dress myself’.

Bold values indicate significant differences.

Considering age, the prevalence of sputum production increased by age and more than one in five subjects older than 60 years reported this respiratory symptom. Both recurrent wheeze and persistent wheeze were more common among subjects older than 40 years. In contrast, the prevalence of both wheeze with breathlessness and asthmatic wheeze declined by age, being more common among subjects aged 40 or younger. The prevalence of dyspnoea also increased significantly by age (Table 2).

### Chronic bronchitis in relation to asthma and COPD

**Fig. 1:**
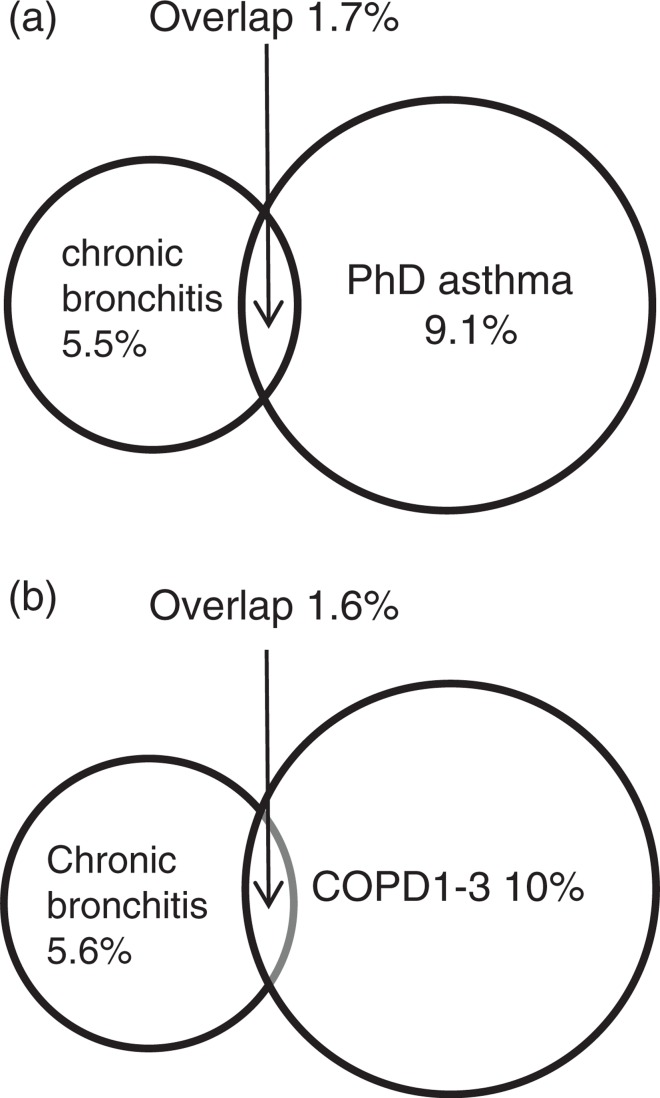
(a) Overlap between physician-diagnosed asthma (PhD asthma) and chronic bronchitis. In the total sample, the prevalence of PhD asthma was 10.8% and the prevalence of chronic bronchitis was 7.2% in the total sample. (b) Overlap between chronic bronchitis and chronic obstructive pulmonary disease (COPD). In the total sample, the prevalence of COPD based on GOLD criteria was 11.6% and the prevalence of chronic bronchitis was 7.2%.

**Fig. 2:**
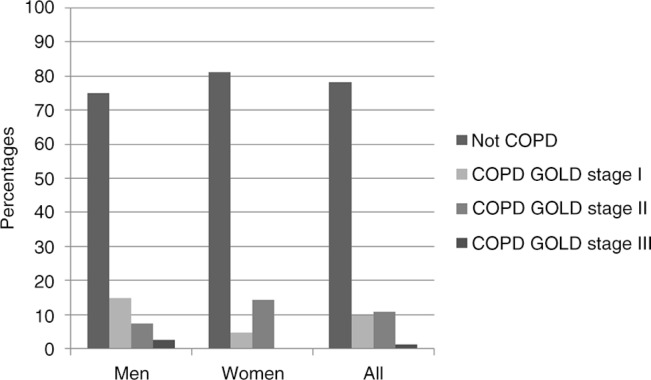
Prevalence of COPD by severity grades among subjects with chronic bronchitis.

There was an overlap, however small, between chronic bronchitis and both reported physician-diagnosed asthma (Fig. 1a) and COPD defined using spirometry (Fig. 1b). The severity of COPD among subjects with chronic bronchitis showed different patterns among men and women, GOLD stage II being more common among women and GOLD stage I among men. Additionally, severe COPD, that is, GOLD stage III, was found only among men, and no one had COPD stage IV (Fig. 2).

### Prevalence of chronic bronchitis by demographic variables, smoking habits and pack-years

The prevalence of chronic bronchitis was higher among subjects living in the city of Gothenburg than among subjects living outside this city. The prevalence declined with increasing level of education but increased by increasing pack-years. Among women, the prevalence of chronic bronchitis increased significantly by increasing numbers of pack-years, and the highest prevalence of chronic bronchitis (21.5%) was observed among ever-smoking women with 20 or more pack-years. Chronic bronchitis was also more common among subjects who had been exposed to occupational dust, gas or fumes compared to subjects who had not been exposed (Table 3).

**Table 3:** Prevalence (%) of chronic bronchitis by area of domicile, level of education, current smoking status, smoking exposure using pack-years, and occupational exposure to dust, gas or fumes in men and women and in three age groups

	Age group (years)				Sex			
			
Variables	≤40	>40 ≤60	>60	Difference^a^ (*p*)	Men	Women	Difference^b^ (*p*)	All
Area of domicile								
Gothenburg	5.4	10.8	9.8	0.071	9.8	7.6	0.332	8.6
Västra Götaland	1.9	5.2	7.6	0.12	4.9	5.7	0.697	5.3
*p*-values	0.25	**0.029**	0.46		**0.035**	0.42		**0.039**
Level of education								
Lower than university	6.7	11.8	9.0	0.28	8.6	10.7	0.40	9.6
University	2.8	4.5	8.3	0.077	6.3	3.7	0.17	4.8
*p*-values trend	0.10	**0.004**	0.85		0.41	**0.001**		**0.001**
Current smoking status								
Non-smokers	2.3	6.4	6.9	0.060	6.1	4.0	0.33	5.0
Ex-smokers	7.1	7.0	7.9	0.97	9.0	5.9	0.26	7.4
Smokers	7.7	15.0	20.6	0.13	8.9	15.9	0.19	13.0
*p*-values trend	0.06	0.054	0.052		0.31	**0.001**		**0.001**
Pack-years among ever smokers								
≤10 years	7.7	7.0	6.1	0.95	8.8	5.5	0.33	6.9
>10 <20 years	22.2	7.3	5.9	0.24	2.6	10.4	0.27	7.8
≥20 years	100	18.2	15.0	0.18	13.4	21.5	0.27	17.0
*p*-values trend	0.025	0.057	0.137		0.389	**0.001**		**0.002**
Exposure to occupational dust, gas or fumes								
No	3.5	5.9	9.2	**0.024**	6.1	6.2	1.00	6.2
Yes	7.0	13.4	7.8	0.29	10.3	8.6	0.83	9.7
*p*-values trend	0.22	**0.014**	0.83		0.092	0.37		**0.046**

a By using Mantel–Haenszel test for trend

b by using Fisher's exact test.

Bold values indicate significant differences.

The differences between men and women exemplified in Table 3 resulted in separate risk analyses, both un-adjusted (Supplementary file 1) and adjusted (Supplementary file 2). These analyses showed that both smoking and low level of education were stronger risk factors for chronic bronchitis among women than men, while area of domicile was a risk factor for chronic bronchitis among men.

### Risk factors for chronic bronchitis and respiratory symptoms

In un-adjusted analyses, the strongest risk factors for chronic bronchitis were 20 or more pack-years and current smoking. Increasing age, lower level of education, 20 or more pack-years among ever smokers were significantly associated with chronic bronchitis, sputum production, recurrent wheeze and dyspnoea. Exposure to occupational dust, gas or fumes was associated with increased risk for chronic bronchitis, sputum production and recurrent wheeze. Area of domicile was only associated with chronic bronchitis (Supplementary file 3).

Multivariate models identified current smoking and age over 60 years to be the strongest risk factors for chronic bronchitis. Increasing age was a significant risk factor for sputum production, dyspnoea and recurrent wheeze (Table 4). When using pack-years instead of current smoking habits, 20 or more pack-years was a significant risk factor for chronic bronchitis (OR 2.72, 95% CI: 1.43–5.16), sputum production (OR 3.37, 95% CI: 2.11–5.36), dyspnoea (OR 2.61, 95% CI: 1.20–5.66) and for recurrent wheeze (OR 3.51, 95% CI: 1.99–6.20) in multivariate models adjusted for age, area of domicile, education level and exposure to occupational dust, gas or fumes, and the other independent variables remained stable; however, age lost its significance (Supplementary file 4).

**Table 4:** Risk factors for chronic bronchitis and respiratory symptoms by using multiple logistic regression analysis

Independent variables		Dependent variables			
	
Variables	Categories	Chronic bronchitis	Sputum production	Dyspnoea grade ≥2^a^	Recurrent wheeze
Age	≤40 years >40 ≤60 years >60 years	1 **2.02 (1.06–3.83)** **2.31 (1.17–4.54)**	1 **2.02 (1.26–3.26)** **3.41 (2.09–5.58)**	1 2.10 (0.79–5.58) **4.38 (1.68–11.44)**	1 **1.95 (1.10–3.43)** **2.14 (1.17–3.94)**
Area of domicile	Västra Götaland Gothenburg	1 **1.85 (1.13–3.03)**	1 0.97 (0.70–1.36)	1 **2.38 (1.21–4.69)**	1 0.87 (0.57–1.33)
Level of education	University Lower than university	1 **1.89 (1.14–3.13)**	1 1.32 (0.93–1.88)	1 2.36 (1.16–4.79)	1 **1.61 (1.03–2.53)**
Current smoking status	Non-smoker Ex-smoker Smoker	1 **1.20 (0.69–2.07)** **2.42 (1.33–4.38)**	1 1.36 (0.93–2.00) **2.93 (1.88–4.57)**	1 1.03 (0.51–2.11) 1.64 (0.71–3.79)	1 1.05 (0.63–1.74) **3.48 (2.07–5.84)**
Exposure to occupational dust, gas or fumes	No Yes	1 1.38 (0.84–2.26)	1 **1.80 (1.26–2.56)**	1 1.50 (0.78–2.89)	1 **1.73 (1.11–2.68)**

Risks expressed as odds ratios (OR) with 95% confidence intervals (CI).

a MRC-scale=Medical Research Council breathlessness scale: Grade 2=‘Walk slower than most people my age on the level’ and/or ‘have to stop for breath when walking at my own pace on the level’. Grade 3=‘stop for breath after walking 100 yards on level ground’. Grade 4=‘get out of breath when I wash myself or dress myself’. Significant risk factors are depicted in bold.

**Fig. 3:**
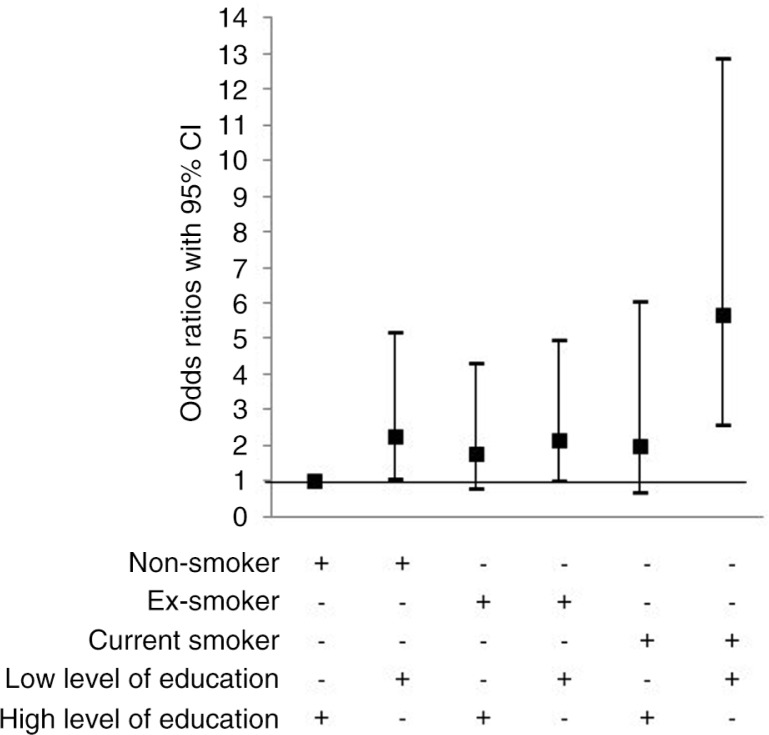
Risk factors for chronic bronchitis by multiple logistic regressions analysis adjusted for age, area of domicile and exposure to occupational dust, gas or fumes. Odds ratios (OR) with 95% confidence intervals (CI). References category: non-smokers with high level of education, i.e. university, as reference category.

**Fig. 4:**
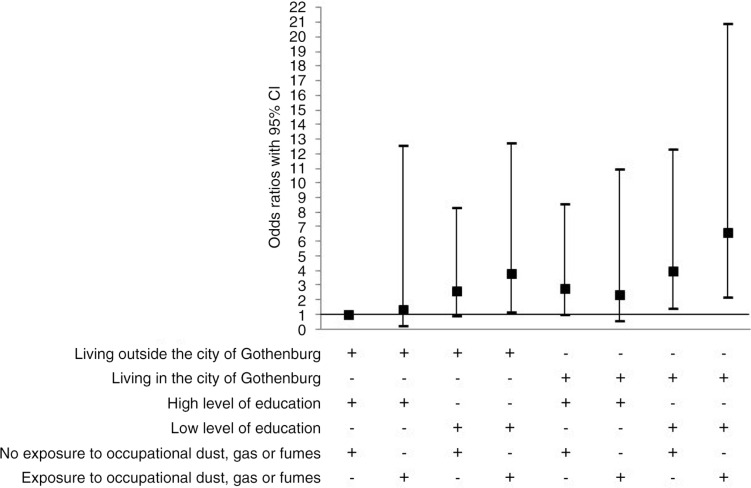
Multivariate logistic regressions based on the total sampled adjusted for age and current smoking status showing odds ratios with 95% confidence intervals (CI) with chronic bronchitis as dependent variable and a combined variable with area of domicile living outside the city of Gothenburg in the region of West Gothia, high level of education, i.e. university and unexposed to occupational dust, gas or fumes as reference category.

A multivariate model using a combined variable method showed that low level of education both in interaction with non-smoking and current smoking yielded an increased risk for chronic bronchitis (Fig. 3). Furthermore, the combination of living in the city of Gothenburg, having low level of education and being exposed to occupational dust, gas or fumes yielded the highest OR for chronic bronchitis. However, living in the city of Gothenburg and having low level of education without exposure to occupational dust, gas or fumes did also increase the risk for chronic bronchitis (Fig. 4). The combination of living outside the city, having low level of education and being exposed to occupational dust, gas or fumes did also result in an increased risk for chronic bronchitis (Fig. 4).

## Discussion

The estimated prevalence of chronic bronchitis was 7.2% being somewhat higher in men (7.6%) than in women (6.8%), but the difference was not significant. The prevalence of chronic bronchitis increased by age, was strongly related to smoking, was not related to sex and decreased by higher level of education. Furthermore, living in the city of Gothenburg was also a risk factor for chronic bronchitis. In particular, living in the city of Gothenburg in combination with low level of education and exposure to occupational dust, gas or fumes increased the risk for chronic bronchitis. However, living outside the city, exposure to occupational dust, gas or fumes in combination with low level of education was also associated with an increased risk for chronic bronchitis. Prevalence based on symptoms was twice as high as based on self-reported, physician-diagnosed chronic bronchitis, which indicates major underdiagnoses.

**Fig. 5:**
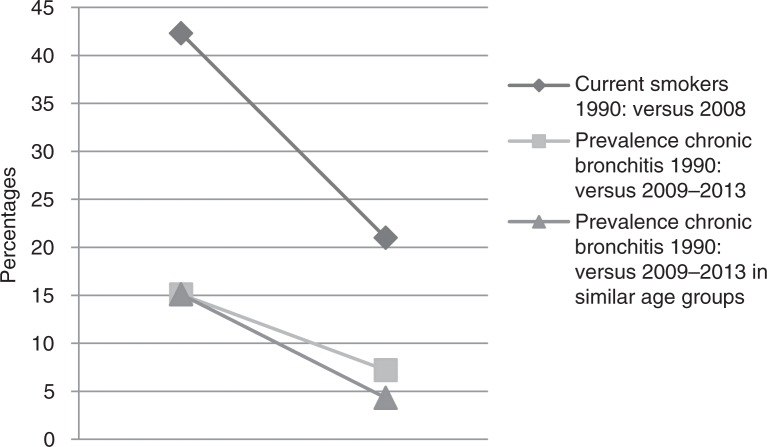
Prevalence trends for chronic bronchitis and current smokers over the last two decades in West Sweden.

Our study suggests that the prevalence of chronic bronchitis in West Sweden has decreased over the past 30 years. The prevalence, 7%, was slightly lower compared to previously reported prevalence of 9% in the 1980s in Northern Sweden (17) and 16% in participants 65 years and older in the 1990s in Denmark (15), while in our study the prevalence was about 9% in subjects older than 60 years. The prevalence of chronic bronchitis tends to have decreased parallel to a decrease in current smoking in recent decades in West Sweden, as illustrated in Fig. 5. In the 1990s, the prevalence of smoking was 42% in the population aged 20–44 (41) compared to 21% in the same age group in 2008 (30) in this geographical area. Figure 5 also shows a decline in prevalence of chronic bronchitis during the same time period: in the 1990s the prevalence was 15% in participants aged 20–44 years (41) compared to 7.2% in the total sample and 4.3% in participants younger than 40 years in the current study. These comparisons and that chronic bronchitis is strongly related to smoking suggest that the prevalence of chronic bronchitis has declined in other parts of Sweden as well because current smoking is less common today compared to a few decades ago (29, 30).

In the recently performed RHINE, which is the corresponding North European part of the ECRHS II with centres in Sweden, Norway, Denmark, Iceland and Estonia, the average prevalence of chronic bronchitis was even lower, 5.4%; however, all participants were younger than 60 years (19). In neighbouring Finland, where the smoking prevalence is higher than in Sweden but with similar social structure, a recent study of prevalence resulted in a higher estimate, namely 14% (28). Similarly in Italy, the prevalence of chronic bronchitis seems to be higher, 12% (27). Nevertheless, notably the known variation in prevalence rates can be attributed to methodological discrepancies, such as differences in the age distribution of the studied samples and use of self-administrated questionnaires versus interviews, where self-administrated questionnaires may result in lower estimates of prevalence (17, 24, 33). The latter has also been demonstrated in studies in Northern Sweden (17, 33) and Northern Finland (42, 43), with self-administrated questionnaires yielding lower prevalence than based on interview.

In contrast to previous studies, we found no difference in prevalence of chronic bronchitis between men and women. An example is the results from Harjavalta, Finland, in the 1960s, where 28% of men had chronic bronchitis versus 6% among women, results that parallels to smoking habits (25). Smoking was considerably more common in men than in women for a long time; however, during the past 2–3 decades, this difference has levelled off and for instance in Sweden today more women than men are smokers. In Sweden in the 1980s about one third of both men and women were smokers (33), while 20 years later the prevalence of smokers who smoked at least once a week was similar, 19%, in both West Sweden and Northern Sweden and more women than men were smokers in the two areas (29, 30). In Finland, chronic bronchitis seems to be still more common in men than in women (28). In line with previous studies (17, 18), increasing age was one determinant of chronic bronchitis.

As mentioned, an explanation for the lower prevalence of chronic bronchitis in the current study compared to previous Scandinavian prevalence rates of chronic bronchitis or chronic or recurrent symptoms of bronchitis (13, 17, 23, 41, 44, 45) could probably be due to a major reduction in smoking in Sweden over the past three decades (29, 30). A similar decreasing smoking trend has been reported in Finland and the decline in prevalence of chronic bronchitis could at least partly be explained by the decrease in smoking (28). The prevalence trends of chronic bronchitis in an Italian study remained fairly stable despite a reduction in smoking (27). Instead, there was a stronger association between chronic bronchitis and lower socio-economic status measured as unemployment/premature retirement (27). This is in accordance with the current study, showing that lower education as proxy for lower socio-economic status was a significant determinant for chronic bronchitis. Additionally, we could show that lower socio-economic class in combination with current smoking among both men and women increased the likelihood for chronic bronchitis. Importantly, the prevalence of chronic bronchitis was higher among current smoking women and among women with 20 or more pack-years, which indicates that women are more susceptible to tobacco smoking as there were no significant differences in smoking habits in our study sample. Chronic bronchitis was also more common among women with a lower level of education, while among men chronic bronchitis was not significantly more common among subjects with lower level of education. The difference between men and women regarding smoking and lower level of education was verified by adjusted risk analyses.

Moreover, we found an area of domicile to be associated with chronic bronchitis as living in the city of Gothenburg was associated with an increased risk with living outside the urbanised area as reference. Living in the city of Gothenburg was also a significant risk factor for dyspnoea. While this could be due to the impact of air pollution, the potential association is controversial, with studies showing conflicting results (21, 46). We could also show that the risk of having chronic bronchitis was a combination of living in the city and lower level of education particularly when exposed to occupational dust, gas or fumes. Further research considering the combined impact of occupational exposure and air pollution is warranted.

The current study showed expected overlaps between chronic bronchitis and asthma, but the overlap between chronic bronchitis and COPD was somewhat less than expected. As previous Scandinavian studies have found a greater overlap between chronic bronchitis and COPD (43, 47), the large decrease in smoking prevalence may have altered phenotypes of both chronic bronchitis and COPD. It could therefore be argued whether chronic bronchitis is a correct wording in these cases. Perhaps, asthma with chronic bronchitis and COPD with chronic bronchitis would be more accurate.

Strengths of the current study were the randomly selected sample from the general population and the sample size, together with a study of non-response (31). Although smokers were somewhat more common among the non-responders, no significant differences in symptom prevalence were found between participants and non-participants. Thus, we conclude our results to be representative for the investigated population. A further strength was that the definition of chronic bronchitis was based on reported symptoms instead of self-reports on physician diagnosis. Another strength was that the definition of chronic bronchitis was based on data collected through structured interviews, which are more detailed than postal questionnaires. Although the interview questionnaires that were used in the current study are widely used and have been validated recently (38), a potential weakness might be recall bias as regards the reports on pack-years. The use of the GLI normal values may have resulted in a low proportion of subjects with moderate and severe COPD as healthy middle-aged and elderly Swedes have higher FEV_1_ and FVC than those suggested by GLI (48). Another weakness might be that the variable exposure of occupational dust, gas or fumes did not contain any information about what kind of exposures these subjects had been exposed to. Because no person reported outcome measure such as health-related quality of life was included in the study, which could be seen as a shortcoming, the current study suggests that future research in this area explores chronic bronchitis in relation to self-reported health outcome.

In conclusion, the estimated prevalence of chronic bronchitis was lower than previously reported estimations from West Sweden, suggesting a decrease in prevalence, which may be due to a reduction in tobacco smoking during the last decades. The current study suggests that chronic bronchitis is a condition related to smoking and social class, and current smoking in combination with lower level of education further increased the risk for this condition.

## Supplementary Material

Chronic bronchitis in West Sweden – a matter of smoking and social class

## References

[1] Fletcher CM, Gilson JG, Hugh-Jones P, Scadding JG (1959). Terminology, definitions and classification of chronic pulmonary emphysema and related conditions. Thorax.

[2] American Thoracic Society (1995). Standards for the diagnosis and care of patients with chronic obstructive pulmonary disease. Am J Respir Crit Care Med.

[3] BTS guidelines for the management of chronic obstructive pulmonary disease (1997). The COPD Guidelines Group of the Standards of Care Committee of the BTS. Thorax.

[4] Siafakas NM, Vermeire P, Pride NB, Yernault JC, Decramer M, Higenbottam T (1995). Optimal assessment and management of chronic obstructive pulmonary disease (COPD). The European Respiratory Society Task Force. Eur Respir J.

[5] Kim V, Han MK, Vance GB, Make BJ, Newell JD, Hokanson JE (2011). COPDGene Investigators. The chronic bronchitic phenotype of COPD: an analysis of the COPDGene Study. Chest.

[6] Corhay L, Vincken W, Schlesser M, Bossuyt P, Imschoot J (2013). Chronic bronchitis in COPD patients is associated with increased risk of exacerbations: a cross-sectional multicentre study. Int J Clin Pract.

[7] Global Initiative for Chronic Obstructive Pulmonary Disease (GOLD). Global strategy for the diagnosis, management, and prevention of chronic obstructive pulmonary disease (2014). http://www.goldcopd.com/.

[8] Lindberg A, Eriksson B, Larsson LG, Rönmark E, Sandström T, Lundbäck B (2006). 7-year cumulative incidence of COPD in an age-stratified general population sample. Chest.

[9] de Marco R, Accordini S, Cerveri I, Corsico A, Antó JM, Künzli N (2007). Incidence of chronic obstructive pulmonary disease in a cohort of young adults according to the presence of chronic cough and phlegm. Am J Respir Crit Care Med.

[10] De Miguel Díez J, Barrera VH, Maestu LP, Garrido PC, García TG, García RJ (2011). Prevalence of anxiety and depression among chronic bronchitis patients and the associated factors. Respirology.

[11] Kanervisto M, Saarelainen S, Vasankari T, Jousilahti P, Heistaro S, Heliövaara M (2010). COPD, chronic bronchitis and capacity for day-to-day activities: negative impact of illness on the health-related quality of life. Chron Respir Dis.

[12] Pelkonen M, Notkola I-L, Nissinen A, Tukiainen H, Koskela H (2006). Thirty-year cumulative incidence of chronic bronchitis and COPD in relation to 30-year pulmonary function and 40-year mortality: a follow-up in middle-aged rural men. Chest.

[13] Vestbo J, Prescott E, Lange P (1996). Association of chronic mucus hypersecretion with FEV1 decline and chronic obstructive pulmonary disease morbidity. Copenhagen City Heart Study Group. Am J Respir Crit Care Med.

[14] Guerra S, Sherrill DL, Venker C, Ceccato CM, Halonen M, Martinez FD (2009). Chronic bronchitis before age 50 years predicts incident airflow limitation and mortality risk. Thorax.

[15] Lange P, Parner J, Prescott E, Vestbo J (2003). Chronic bronchitis in an elderly population. Age Ageing.

[16] Ekberg-Aronsson M, Pehrsson K, Nilsson J-Å, Nilsson PM, Löfdahl C-G (2005). Mortality in GOLD stages of COPD and its dependence on symptoms of chronic bronchitis. Respir Res.

[17] Lundbäck B, Stjernberg N, Nyström L, Lundbäck K, Lindström M, Rosenhall L (1993). An interview study to estimate prevalence of asthma and chronic bronchitis. The obstructive lung disease in northern Sweden study. Eur J Epidemiol.

[18] Ferré A, Fuhrman C, Zureik M, Chouaid C, Vergnenègre A, Huchon G (2012). Chronic bronchitis in the general population: influence of age, gender and socio-economic conditions. Respir Med.

[19] Holm M, Kim J-L, Lillienberg L, Storaas T, Jögi R, Svanes C (2012). Incidence and prevalence of chronic bronchitis: impact of smoking and welding. The RHINE study. Int J Tuberc Lung Dis.

[20] Meteran H, Thomsen SF, Harmsen L, Ohm Kyvik K, Skytthe A, Backer V (2012). Risk of chronic bronchitis in twin pairs discordant for smoking. Lung.

[21] Viegi G, Pedreschi M, Baldacci S, Chiaffi L, Pistelli F, Modena P (1999). Prevalence rates of respiratory symptoms and diseases in general population samples of North and Central Italy. Int J Tuberc Lung Dis.

[22] Lindgren A, Stroh E, Montnémery P, Nihlén U, Jakobsson K, Axmon A (2009). Traffic-related air pollution associated with prevalence of asthma and COPD/chronic bronchitis. A cross-sectional study in Southern Sweden. Int J Health Geogr.

[23] Bakke P, Eide O, Hanoa R, Gulsvik A (1991). Occupational dust or gas exposure and prevalences of respiratory symptoms and asthma in a general population. Eur Respir J.

[24] Halbert RJ, Natoli JL, Gano A, Badamgarav E, Buist AS, Mannino DM (2006). Global burden of COPD: systematic review and meta-analysis. Eur Respir J.

[25] Huhti E (1965). Prevalence of respiratory symptoms, chronic bronchitis and pulmonary emphysema in a Finnish rural population. Field survey of age group 40–64 in the Harjavalta area.

[26] Ehrlich RI, White N, Norman R, Laubscher R, Steyn K, Lombard C (2004). Predictors of chronic bronchitis in South African adults. Int J Tuberc Lung Dis.

[27] Accordini S, Corsico AG, Cerveri I, Antonicelli L, Attena F, Bono R (2013). Diverging trends of chronic bronchitis and smoking habits between 1998 and 2010. Respir Res.

[28] Pelkonen MK, Notkola I-L, Laatikainen TK, Koskela HO (2014). Twenty-five year trends in prevalence of chronic bronchitis and the trends in relation to smoking. Respir Med.

[29] Backman H, Hedman L, Jansson S-A, Lindberg A, Lundbäck B, Rönmark E (2014). Prevalence trends in respiratory symptoms and asthma in relation to smoking – two cross-sectional studies ten years apart among adults in northern Sweden. World Allergy Organ J.

[30] Lötvall J, Ekerljung L, Rönmark EP, Wennergren G, Lindén A, Rönmark E (2009). West Sweden Asthma Study: prevalence trends over the last 18 years argues no recent increase in asthma. Respir Res.

[31] Rönmark EP, Ekerljung L, Lötvall J, Torén K, Rönmark E, Lundbäck B (2009). Large scale questionnaire survey on respiratory health in Sweden: effects of late- and non-response. Respir Med.

[32] Ekerljung L, Bjerg A, Bossios A, Axelsson M, Torén K, Wennergren G (2014). Five-fold increase in use of inhaled corticosteroids over 18 years in the general adult population in West Sweden. Respir Med.

[33] Lundbäck B, Nyström L, Rosenhall L, Stjernberg N (1991). Obstructive lung disease in northern Sweden: respiratory symptoms assessed in a postal survey. Eur Resp J.

[34] Pallasaho P, Lundbäck B, Läspä S-L, Jönsson E, Sovijärvi A, Laitinen L (1999). Increasing prevalence of asthma but not chronic bronchitis in Finland – report from the FinEsS-Helsinki study. Respir Med.

[35] Meren M, Jannus-Pruljan L, Loit H-M, P[otilde]lluste J, Jönsson E, Kiviloog J (2001). Asthma, chronic bronchitis and respiratory symptoms among adults in Estonia. Respir Med.

[36] Kotaniemi J, Lundbäck B, Nieminen M, Sovijärvi A, Laitinen L (2001). Increase of asthma in adults in Northern Finland – a report from The FinEsS study. Allergy.

[37] Lâm HT, Rönmark E, Tu’ò'ng NV, Ekerljung L, Chúc NT, Lundbäck B (2011). Increase in asthma and a high prevalence of bronchitis: results from a population study among adults in urban and rural Vietnam. Respir Med.

[38] Ekerljung L, Rönmark E, Lötvall J, Wennergren G, Torén K, Lundbäck B (2013). Questionnaire layout and wording influence prevalence and risk estimates of respiratory symptoms in a population cohort. Clin Respir J.

[39] Quanjer PH, Stanojevic S, Cole TJ, Baur X, Hall GL, Culver BH (2012). Multi-ethnic reference values for spirometry for the 3-95-yr age range: the global lung function 2012 equations. Eur Respir J.

[40] Bestall JC, Paul EA, Garrod R, Garnham R, Jones PW, Wedzicha JA (1999). Usefulness of the Medical Research Council (MRC) dyspnoea scale as a measure of disability in patients with chronic obstructive pulmonary disease. Thorax.

[41] Björnsson E, Plaschke P, Norrman E, Janson C, Lundbäck B, Rosenhall A (1994). Symptoms related to asthma and chronic bronchitis in three areas of Sweden. Eur Respir J.

[42] Lindström M, Kotaniemi J, Jönsson E, Lundbäck B (2001). Smoking, respiratory symptoms and diseases – a comparative study between Northern Sweden and Northern Finland – report from the FinEsS study. Chest.

[43] Kotaniemi JT, Sovijärvi A, Lundbäck B (2005). Chronic obstructive pulmonary disease in Finland: prevalence and risk factors. COPD.

[44] Larsson L, Boethius G, Uddenfeldt M (1993). Differences in utilization of asthma drugs between two neighbouring Swedish provinces: relation to symptom reporting. Eur Respir J.

[45] Montnemery P, Ädelroth E, Heuman K, Johannisson A, Johansson S-Å, Lindholm L-H (1998). Prevalence of obstructive lung diseases and respiratory symptoms in southern Sweden. Respir Med.

[46] Cai Y, Schikowski T, Adam M, Buschka A, Carsin A-E, Jacquemin B (2014). Cross-sectional associations between air pollution and chronic bronchitis: an ESCAPE meta-analysis across five cohorts. Thorax.

[47] Lundbäck B, Lindberg A, Lindström M, Rönmark E, Jonsson A-C, Jönsson E (2003). Not 15 but 50% of smokers develop COPD? – Report from the Obstructive Lung Disease in Northern Sweden Studies. Respir Med.

[48] Backman H, Lindberg A, Sovijärvi A, Larsson K, Lundbäck B, Rönmark E (2015). Evaluation of the global lung function initiative 2012 reference values for spirometry in a Swedish population sample. BMC Pulm Med.

