# Supplementary material for: Chronic bronchitis in West Sweden – a matter of smoking and social class
Source: Eur Clin Respir J. 2016 Jul 13;3:10.3402/ecrj.v3.30319. doi: 10.3402/ecrj.v3.30319 (PMC4947195; doi:10.3402/ecrj.v3.30319)
Supplement: Chronic bronchitis in West Sweden – a matter of smoking and social class [file ECRJ-3-30319-s001.docx]

**Supplementary file 1. Unadjusted risk factors of chronic bronchitis in men and women respectively. Risks expressed as odds ratios (OR) with 95% confidence intervals (CI).**

| **Independent variables** | | **Chronic bronchitis** | |
| --- | --- | --- | --- |
| Variables | Categories | Men | Women |
| Age | ≤40 years  >40 ≤60 years  >60 years | 1  1.84 (0.74-4.58)  2.04 (0.83-5.03) | 1  2.05 (0.89-4.70)  2.13 (0.86-5.28) |
| Area of domicile | Västra Götaland  Gothenburg | **1**  **2.10 (1.05-4.21)** | 1  1.35 (0.70-2.57) |
| Level of education | University  High school  Secondary school or less | 1  1.26 (0.61-2.58)  1.74 (0.73-4.13) | **1**  **3.26 (1.60-6.63)**  **2.77 (1.11-6.91)** |
| Level of education | University  Lower than university | 1  1.39 (0.72-2.69) | 1  **3.11 (1.59-6.09)** |
| Current smoking status | Non-smoker  Ex-smoker  Smoker | 1  1.54 (0.77-3.10)  1.51 (0.60-3.80) | 1  1.49 (0.67-3.34)  **4.50 (2.09-9.68)** |
| Pack-years | Non-smoker  ≤10 years  >10 <20 years  ≥ 20 years | 1  1.50 (0.66-3.43)  0.42 (0.05-3.25)  **2.40 (1.07-5.41)** | 1  1.38 (0.55-3.45)  **2.75 (1.08-7.00)**  **6.52 (2.85-14.90)** |
| Pack-years among ever smokers | ≤10 years  >10 <20 years  ≥ 20 years | 1  0.28 (0.03-2.25)  1.60 (0.64-3.96) | 1  2.00 (0.72-5.56)  **4.74 (1.88-11.96)** |
| Exposure to occupational dust, gas or fumes | No  yes | 1  1.07 (0.46-2.48) | 1  1.28 (0.52-3.12) |

Significant factors are depicted in bold

**Supplementary file 2. Risk factors for chronic bronchitis in men and women respectively by using multiple logistic regression analysis. Risks expressed as odds ratios (OR) with 95% confidence intervals (CI).**

| **Independent variables** | | **Chronic bronchitis** | |
| --- | --- | --- | --- |
| Variables | Categories | Men | Women |
| Age | ≤40 years  >40 ≤60 years  >60 years | 1  2.02 (0.73-5.62)  1.93 (0.68-5.52) | 1  1.07 (0.42-2.71)  0.95 (0.34-2.64) |
| Area of domicile | Västra Götaland  Gothenburg | **1**  **2.44 (1.15-5.15)** | 1  1.44 (0.72-2.89) |
| Level of education | University  Lower than university | 1  1.29 (0.60-2.78) | 1  **2.75 (1.29-5.87)** |
| Pack-years | Non-smoker  ≤10 years  >10 <20 years  ≥ 20 years | 1  1.22 (0.52-2.86)  0.34 (0.04-2.71)  1.59 (0.65-3.89) | 1  1.39 (0.54-3.58)  2.40 (0.89-6.48)  **4.74 (1.85-12.15)** |
| Exposure to occupational dust, gas or fumes | No  yes | 1  1.59 (0.78-3.27) | 1  1.061 (0.45-2.52) |

**Supplementary file 3. Unadjusted risk factors for chronic bronchitis and respiratory symptoms. Risks expressed as odds ratios (OR) with 95% confidence intervals (CI).**

| **Independent variables** |  | **Dependent variables** | | | |
| --- | --- | --- | --- | --- | --- |
| Variables | Categories | Chronic bronchitis | Sputum production | Dyspnea grade ≥ 2* | Recurrent wheeze |
| Sex | Men  Women | 1  0.90 (0.57-1.40) | 1  0.81 (0.59-1.11) | 1  1.39 (0.75-2.57) | 1  0.96 (0.64-1.43) |
| Age | ≤40 years  >40 ≤60 years  >60 years | 1  **1.95 (1.05-3.60)**  **2.12 (1.12-4.0)** | 1  **2.06 (1.31-3.24)**  **3.2 (2.04-5.06)** | 1  1.89 (0.72-4.91)  **4.10 (1.65-10.19)** | 1  **1.90 (1.11-3.26)**  **1.90 (1.08-3.35)** |
| Area of domicile | Västra Götaland  Gothenburg | 1  **1.66 (1.03-2.67)** | 1  0.90 (0.66-1.23) | 1  1.86 (0.96-3.59) | 1  0.84 (0.56-1.25) |
| Level of education | University  High school  Secondary school or less | 1  **2.07 (1.25-3.43)**  **2.24 (1.19-4.19)** | 1  **1.74 (1.22-2.47)**  **2.16 (1.39-3.36)** | 1  **2.19 (1.04-4.59)**  **4.40 (2.00-9.72)** | 1  **1.97 (1.24-3.12)**  **2.74 (1.59-4.73)** |
| Level of education | University  Lower than university | 1  **2.12 (1.33-3.39)** | 1  **1.86 (1.34-2,57)** | 1  **2.80 (1.43-5.50)** | 1  **2.19 (1.43-3.34)** |
| Current smoking status | Non-smoker  Ex-smoker  Smoker | 1  1.52 (0.90-2.58)  **2.85 (1.62-5.02)** | 1  **1.82 (1.26-2.62)**  **2.92 (1.92-4.44)** | 1  1.55 (0.78-3.08)  1.89 (0.84-4.24) | 1  1.37 (0.84-2.24)  **3.82 (2.33-6.28)** |
| Pack-years | Non-smoker  ≤10 years  >10 <20 years  ≥ 20 years | 1  1.42 (0.77-2.62)  1.62 (0.74-3.52)  **3.90 (2.20-6.93)** | 1  **1.80 (1.19-2.73)**  1.44 (0.80-2.57)  **4.95 (3.23-7.60)** | 1  0.67(0.24-1.86)  1.61 (0.58-4.49)  **4.56 (2.24-9.28)** | 1  1.30 (0.73-2.30)  1.32 (0.61-2.82)  **4.84 (2.90-8.10)** |
| Pack-years among ever smokers | ≤10 years  >10 <20 years  ≥ 20 years | 1  1.14 (0.49-2.61)  **2.74 (1.44-5.22)** | 1  0.80 (0.44-1.46)  **2.76 (1.74-4.37)** | 1  2.39 (0.68-8.43)  **6.77 (2.44-18.78)** | 1  1.01 (0.45-2.30)  **3.73 (2.06-6.76)** |
| Exposure to occupational dust, gas or fumes | No  yes | **1**  **1.64 (1.02-2.64)** | **1**  **1.99 (1.42-2.79)** | 1  1.79 (0.95-3.36) | **1**  **2.01 (1.32-3.06)** |

*****MRC-scale=Medical Research Council breathlessness scale: Grade 2=”Walk slower than most people my age on the level” and/or “have to stop for breath when walking at my own pace on the level”. Grade 3= “stop for breath after walking 100 yards on level ground”

Grade 4=” get out of breath when I wash myself or dress myself” Significant risk factors are depicted in bold.

**Supplementary file 4. Risk factors for chronic bronchitis by using multiple logistic regression analysis. Risks expressed as odds ratios (OR) with 95% confidence intervals (CI).**

| **Independent variables** |  | **Dependent variables** | | | |
| --- | --- | --- | --- | --- | --- |
| Variables | Categories | Chronic bronchitis | Sputum production | Dyspnea grade ≥2* | Recurrent wheeze |
| Age | ≤40 years  <40 ≤60 years  >60 years | 1  1.45 (0.74-2.87)  1.30 (0.63-2.68) | 1  1.58 (0.96-2.62)  **2.19 (1.31-3.66)** | 1  1.70 (0.58-4.97)  **2.93 (1.02-8.46)** | 1  1.55 (0.81-2.94)  1.42 (0.72-2.80) |
| Area of domicile | Västra Götaland  Gothenburg | 1  **1.83 (1.11-3.03)** | 1  0.97 (0.69-1.37) | 1  **2.22 (1.12-4.40)** | 1  0.87 (0.56-1.34) |
| Level of education | University  Lower than university | 1  **2.00 (1.17-3.41)** | 1  1.33 (0.92-1.93) | 1  2.01 (0.97-4.18) | 1  1.40 (0.87-2.25) |
| Pack years | Non-smoker  ≤10 years  >10 <20 years  ≥ 20 years | 1  1.30 (0.70-2.42)  1.36 (0.61-3.04)  **2.72 (1.43-5.16)** | 1  **1.68 (1.10-2.57)**  1.14 (0.63-2.07)  **3.37 (2.11-5.36)** | 1  0.58 (0.21-1.62)  1.14 (0.40-3.28)  **2.61 (1.20-5.66)** | 1  1.17 (0.66-2.09)  1.09 (0.50-2.39)  **3.51 (1.99-6.20)** |
| Exposure to occupational dust, gas or fumes | No  Yes | 1  1.28 (0.76-2.14) | 1  **1.64 (1.13-2.37)** | 1  1.33 (0.67-2.63) | 1  **1.61 (1.01-2.55)** |

*MRC-scale=Medical Research Council breathlessness scale:

Grade 2=”Walk slower than most people my age on the level” and/or “have to stop for breath when walking at my own pace on the level”.

Grade 3= “stop for breath after walking 100 yards on level ground”

Grade 4=” get out of breath when I wash myself or dress myself”. Significant risk factors are depicted in bold.
